# Quantitative 3.0T MR Spectroscopy Reveals Decreased Creatine Concentration in the Dorsolateral Prefrontal Cortex of Patients with Social Anxiety Disorder

**DOI:** 10.1371/journal.pone.0048105

**Published:** 2012-10-23

**Authors:** Qiang Yue, Mengqi Liu, Xiaojing Nie, Qizhu Wu, Jun Li, Wei Zhang, Xiaoqi Huang, Qiyong Gong

**Affiliations:** 1 Huaxi MR Research Center (HMRRC), Department of Radiology, West China Hospital of Sichuan University, Chengdu, People’s Republic of China; 2 Department of Psychiatry, West China Hospital of Sichuan University, Chengdu, People’s Republic of China; 3 Department of geriatrics, West China Hospital of Sichuan University, Chengdu, People’s Republic of China; Bellvitge Biomedical Research Institute-IDIBELL, Spain

## Abstract

**Background:**

The brain biochemical changes of social anxiety have not been clarified although there have been a limited number of MR spectroscopic studies which utilized metabolite/creatine ratios. Present study aimed to explore the alteration of absolute metabolite concentration in social anxiety disorder using quantitative MR spectroscopy.

**Materials and Methods:**

With a 3.0T MR scanner, single voxel MR spectroscopy (stimulated echo acquisition mode, TR/TE/TM = 2000/20/16 ms) was performed in the left dorsolateral prefrontal cortex and related regions of nine medication-free patients with social anxiety disorder and nine controls. Absolute metabolite concentration was calculated using tissue water as the internal reference and corrected for the partial volume of cerebrospinal fluid.

**Results:**

In the left dorsolateral prefrontal cortex, the N-acetyl aspartate/creatine ratio of patients was significantly higher than that of controls, and this was due to the decrease of creatine concentration instead of the increase of N-acetyl aspartate concentration. Furthermore, the creatine concentration of the left dorsolateral prefrontal cortex was negatively correlated with the scores of Liebowitz social anxiety scale.

**Conclusions:**

The alteration of creatine level in the left dorsolateral prefrontal cortex suggests abnormal energy metabolism and correlates with symptom severity in social anxiety disorder. And metabolite concentration is preferable to metabolite/creatine ratio for the investigation of individual, absolute metabolite changes in this region of social anxiety disorder.

## Introduction

Social anxiety disorder (SAD) is among the most common anxiety disorders and is among the most common psychiatric disorders. The prevalence of SAD in China is reported to be 3.2–8.15% [1], [2]. SAD patients experience impairment in their work, home, and social relationships. Moreover, it is reported that 69%–92% of SAD patients have at least one kind of other mental disorders including other anxiety disorders (the most common complication), major depressive disorders, and substance abuse disorders [3].

Magnetic resonance spectroscopy (MRS) allows in-depth investigation of metabolic changes in specific brain regions. Only a few MRS studies of SAD [4]–[8] have been reported, most of which used metabolites/creatine (Cr) ratios to represent absolute metabolite changes based on the assumption that Cr level remained stable under various conditions. Elevated glutamine/Cr in whole brain and the thalamus was reported and regarded as the proof of overactivity of glutamatergic system [4]. Increased choline (Cho)/Cr in cortical gray matter was also reported and interpreted as accelerated activity of phospholipase-C [5]. N-acetyl aspartate (NAA)/Cr was found to be elevated in anterior cingulate cortex (ACC) but reduced in occipital cortex, and NAA/Cr in ACC was positively correlated with the severity of anxiety symptoms [8].

Recent quantitative MRS studies, however, have demonstrated abnormal changes of Cr level in other psychiatric disorders [9]–[11]. In depressive patients NAA, Cho and myo-inositol to Cr ratios were found to be significantly lower than in controls, whereas it was attributed to the increase of Cr concentration instead of the decrease of other metabolite concentrations [12]. Thus it is also necessary to clarify whether Cr is altered in SAD patients. Besides, information concerning other metabolic changes expressed in metabolite ratios (like NAA/Cr, Cho/Cr) also needs to be updated with absolute concentrations. And this is where we set out to perform this quantitative MRS study.

Previous studies revealed functional abnormalities of SAD patients in prefrontal cortex, ACC, and limbic/paralimbic regions which comprised corticolimbic circuitry and participated in the genesis of fear and anxiety [13]–[15]. In addition, thalamus and striatum abnormalities were also found in SAD patients, suggesting that these nuclei may also be involved in the pathogenesis of SAD [16], [17]. Interestingly, the left hemisphere seems to be more associated with fear conditioning [18], [19]. So in the present study we located the Volume of Interests (VOI) in the left dorsolateral prefrontal cortex (DLPFC), ACC, bilateral putamens, and left thalamus.

## Materials and Methods

### Subjects

Nine SAD patients and nine healthy controls from the same community were included in this study. SAD was diagnosed by experienced psychiatrists according to the Diagnostic and Statistical Manual of Mental Disorders-IV (DSM-IV, American Psychiatric Association). To be included, patients must be medication free, and not comorbid with any other psychiatric or neurologic disorders, or any other contraindications to MR imaging. Each subject was evaluated by the psychiatrists with Liebowitz Social Anxiety Scale (LSAS) to assess the severity of symptom. This study was approved by the local ethics committee of West China Hospital, and written informed consent was obtained from each subject.

Demographic characteristics of the participants were summarized in Table 1.

**Table 1:** Demographic characteristics of the participants.

	Sample size	Male/female	Age(years)	Avoidance part of LSAS score	Fear part of LSAS score	Total LSAS score
**SAD**	9	5/4	21.6±2.5	27.3±5.9	30.0±6.3	57.3±11.5
**Controls**	9	5/4	21.2±2.0	14.7±3.2	12.0±5.7	26.7±6.0

### MR Spectroscopy

**Figure 1:**
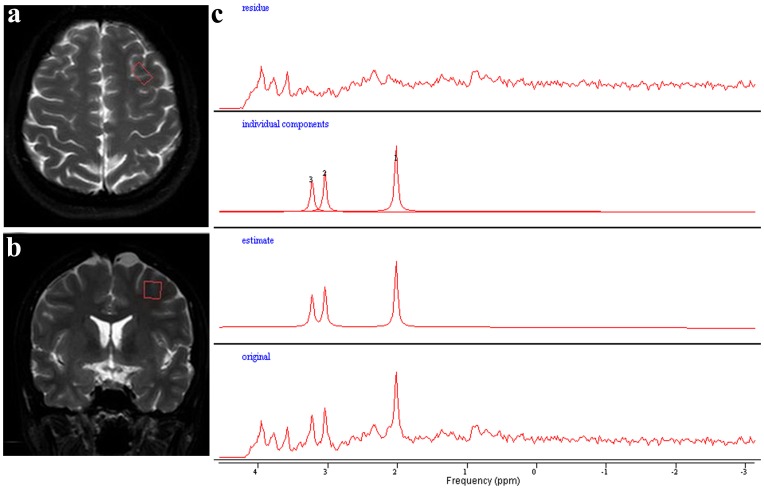
MRS data acquisition and post-processing. (a) and (b) are the transversal and sagittal view of the brain, respectively. The red rectangles indicate the volume of interest representing the left dorsolateral prefrontal cortex (DLPFC) from where the spectrum (c) is acquired. (c) is the spectrum of the left DLPFC. From bottom to the top are the original spectrum, the estimated spectrum yielded by ‘Advanced Method for Accurate, Robust and Efficient Spectral fitting (AMARES)’ package, the individual peak components and the residual spectrum.

All the MRI and MRS examinations were performed on a 3.0 T MR scanner (Philips Achieva, Netherlands). The traditional MR imaging sequences included fast spin-echo T2-weighted images (TR/TE 4000/100 ms) and spin-echo T1-weighted images (TR/TE 500/15 ms) in three orthogonal planes. Single-voxel MR spectroscopy was performed using stimulated echo acquisition mode (STEAM) sequence. Both unsuppressed tissue water and metabolite with water suppression spectra were acquired, with the following parameters: TR/TE/TM = 2000/20/16 ms, spectral bandwidth = 2000 Hz, data points = 1024, number of signals averaged = 128 for metabolites and tissue water, and scanning time = 4 minutes and 56 seconds. VOI was placed in the left DLPFC (Fig. 1a, b), ACC, the left and right putamens and the left thalamus. The Mean±SD VOI volume was 3.5±0.2, 3.4±0.3, 2.8±0.2, 2.9±0.2, 3.2±0.3 cubic centimeters, respectively. Shimming, frequency adjustment, and water suppression were automatically accomplished before data acquisition.

Raw spectral data were exported and processed using jMRUI 3.0 (www.mrui.uab.es/mrui). Phase correction and 3 Hz Lorentzian apodization were first performed, and then tissue water and metabolite (including NAA, Cr and Cho) peak areas were obtained using ‘Advanced Method for Accurate, Robust and Efficient Spectral fitting (AMARES)’ package (Fig. 1c). Prior knowledge including chemical shift (NAA at 2.02 ppm, Cr at 3.03 ppm and Cho at 3.22 ppm with a deviation range of ±0.05 ppm for all), lineshape (Gaussian lineshape was used for all), linewidth (the initial value set for simulation was 4 Hz and was allowed to vary within a range of 1–8 Hz) was incorporated into the fitting algorithm. Soft constraints were applied to all the simulations. And then, absolute metabolite concentration was calculated using tissue water as the internal reference [20]–[22]. Tissue water concentration used was 35 mol/kg wet weight [23]–[25], and the T1 and T2 relaxation constants of tissue water and metabolites were obtained from reported values under the same field strength and the same sequence (STEAM) [26]–[28].

To correct the partial volume effect of cerebrospinal fluid (CSF) contained in the VOIs of ACC and left DLPFC, segmentation of the T2-weighted images was performed as we previously described [29]. The volume of CSF in each slice was calculated by multiplying their areas with slice thickness, and their summation gave total volume of CSF parts within the voxel. We assumed that CSF parts contributed to tissue water rather than to metabolite, and the corrected metabolite concentration was calculated as follows: Corrected metabolite concentration = metabolite concentration × VOI volume/(VOI volume − CSF volume).

### Statistical Analysis

Statistical analysis was performed using the PASW Statistics 18.0 software package (IBM company, USA). The normal distribution and the homogeneity of variance of data were first verified. Then comparisons of metabolite/Cr ratios and each absolute metabolite concentration of the five VOIs between SAD subjects and controls were carried out with independent t-tests, assuming that there was no interaction between different brain regions and metabolites. In each VOI linear correlation analyses were performed between absolute Cr concentration and the total LSAS score, the avoidance part of the LSAS score (the sum of the scores reflecting the avoidance behavior), and the fear part of the LSAS score (the sum of the scores reflecting the fear emotion), respectively. Significance was set at p<0.05.

## Results

The NAA/Cr peak area ratio of the left DLPFC of SAD patients was significantly higher than that of controls. The absolute Cr concentration in the left DLPFC of SAD patients was mildly, but significantly lower than that of controls; while the absolute NAA concentration of the left DLPFC was not significantly different. No significant difference between patients and controls was found for other peak area ratios or absolute concentrations, or in other regions. Detailed absolute metabolite concentrations and metabolite/Cr peak area ratios of each VOI were summarized in Table 2.

**Table 2:** Absolute metabolite concentrations and metabolite/creatine peak area ratios of each volume of interest (VOI).

VOI	Metabolites	Metabolite/Cr Peak Area Ratios and Absolute Concentrations#			
		SAD(mean±SD)	control(mean±SD)	t value	P value
**Left DLPFC**	**NAA** *	15.573±1.571	16.194±1.560	−0.842	0.412
	**Cho** *	2.373±0.597	2.757±0.657	−1.296	0.213
	**Cr** *	11.217±1.297	13.392±2.220	−2.539	**0.022**
	**NAA/Cr**	1.398±0.160	1.230±0.167	2.186	**0.044**
	**Cho/Cr**	0.209±0.034	0.207±0.034	0.149	0.883
**ACC**	**NAA** *	14.839±2.081	15.173±3.564	−0.236	0.817
	**Cho** *	4.186±1.057	3.710±0.929	0.942	0.362
	**Cr** *	14.737±3.223	14.836±2.276	−0.069	0.946
	**NAA/Cr**	1.034±0.184	1.019±0.159	0.176	0.863
	**Cho/Cr**	0.292±0.080	0.253±0.063	1.060	0.307
**Left Putamen**	**NAA**	13.398±1.552	12.871±1.612	0.719	0.482
	**Cho**	2.596±0.483	3.006±0.664	−1.565	0.136
	**Cr**	13.730±1.630	13.834±1.769	−0.132	0.896
	**NAA/Cr**	0.987±0.158	0.950±0.207	0.438	0.667
	**Cho/Cr**	0.191±0.038	0.216±0.027	−1.595	0.129
**Right Putamen**	**NAA**	12.310±1.786	12.809±1.692	−0.608	0.551
	**Cho**	2.164±1.320	2.557±0.735	−0.779	0.447
	**Cr**	13.303±2.234	13.913±2.063	−0.602	0.556
	**NAA/Cr**	0.947±0.198	0.931±0.138	0.191	0.851
	**Cho/Cr**	0.154±0.078	0.184±0.047	−0.977	0.343
**Left Thalamus**	**NAA**	14.960±2.404	14.209±4.071	0.506	0.620
	**Cho**	2.761±0.840	2.293±0.792	1.228	0.236
	**Cr**	10.402±2.136	11.053±3.687	−0.487	0.633
	**NAA/Cr**	1.475±0.296	1.362±0.399	0.715	0.484
	**Cho/Cr**	0.269±0.080	0.215±0.073	1.489	0.155

* Corrected for the partial volume effect of cerebrospinal fluid.

# The unit of the absolute concentration is mol/kg wet weight.

Abbreviations: DLPFC-Dorsal Lateral Prefrontal Cortex; ACC-Anterior Cingulate Cortex.

**Figure 2:**
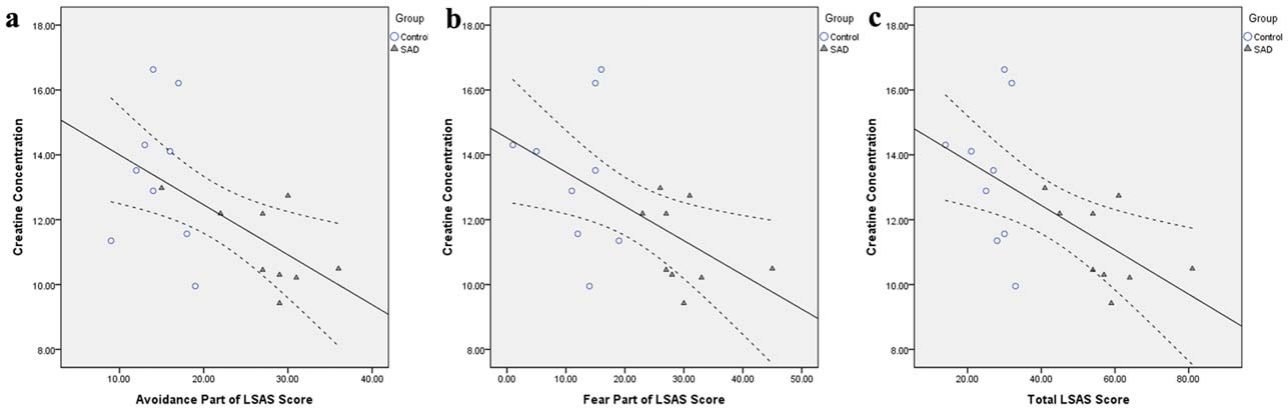
Correlation between creatine concentration and LSAS score. Creatine concentration of the left DLPFC is negatively correlated with the avoidance part of the LSAS score (r = −0.589, p = 0.010) (a), the fear part of the LSAS score (r = −0.553, p = 0.017) (b), and the total LSAS score (r = −0.594, p = 0.007) (c), respectively.

Correlation analysis revealed a significantly negative linear correlation between corrected Cr concentration of the left DLPFC and the avoidance part of the LSAS score (r = −0.589, p = 0.010), the fear part of the LSAS score (r = −0.553, p = 0.017), and the total LSAS score (r = −0.594, p = 0.007), respectively (Fig. 2). No significant correlation was found in other VOIs.

## Discussion

One of our major findings was the mild decrease of Cr concentration in the left DLPFC region, and the latter led to the increased of NAA/Cr ratio although NAA concentration was not significantly changed. Other studies using absolute quantification methods have also demonstrated Cr level, instead of remaining stable as assumed, was actually altered in depressive disorder [9], bipolar disorder [10], and panic disorder [11]. So metabolite/Cr ratio may lead to misinterpretation in case of a changed Cr level. Thus current study demonstrated that absolute metabolite concentration is preferable to metabolite/Cr ratios for unambiguous interpretation of absolute metabolite changes in the left DLPFC of SAD patients.

Cr serves as the reserves of high energy phosphates and as a buffer in adenosine triphosphate and adenosine diphosphate reservoir, and adenosine triphosphate can supply energy for cells to protect tissues from hypoxia-induced damage. Meanwhile, Cr can protect neurons against the toxicity of glutamate and β-amyloid [30], and Cr supplementation enhances brain function under normal and stress conditions [31]. Consistent with our finding, the decrease of Cr level was also reported in the centrum semiovale of patients with generalized anxiety disorder [32] and in the left amygdala of patients with borderline personality disorder (BPD) [9]. Castillo et al. [33], Schaller et al. [34] and Preedy et al. [35] claimed that Cr level was decreased in hypermetabolic states, and this was confirmed in tumors [13], [36] and BPD [9], [10]. Using both ^18^FDG-PET and MRS, previous studies reported simultaneous changes of enhanced ^18^FDG uptake and decreased Cr level in glioma [13] and in cachexia-inducing murine adenocarcinoma [36]. In the amygdalae of patients with BPD, decrease of Cr [9], overactivity [37], and hypermetabolism [10] have all been observed using MRS, functional MR imaging and positron emission tomography (PET), respectively in different studies. Likewise, our finding of decreased Cr level in the DLPFC may also suggest a regional hypermetabolic state. Although evidence currently available is not yet sufficient to establish a direct, causal link, findings from other relevant studies of SAD support this speculation: Tillfors et al. [38] observed increased cerebral blood flow in the same region of SAD patients using PET; Guyer et al. [39] performed functional MRI study and observed increased activity in their ventrolateral prefrontal cortex during provoked anticipatory anxiety in patients with SAD. The hypermetabolism and overactivity of DLPFC is possibly due to the up-regulation of the glutamatergic system. Glutamate is the major excitatory neurotransmitter in the central nervous system and excessive glutamate release is associated with fear-related learning and reactivity [40]. And increased glutamate/glutamine (Glx) level has been reported in the whole brain of SAD patients [4], [8]. Based on our findings and previous reports, a possible pathway for the genesis of SAD may be outlined, in which Cr may play a bridging role. That is, sustained anxiety state causes enhanced excitatory neurotransmitter release; the latter leads to the overactivity and hypermetabolism of DLPFC and subsequently overconsumption of Cr; and the decrease of Cr in turn makes neurons susceptible to the toxicity of glutamate and causes neural damage. However, the hypothesis still needs further studies to provide support particularly for the critical link between low Cr level and hypermetabolic state.

We did not find any inter-group difference of NAA and Cho concentration. In contrast, both Tupler et al. [5] and Phan et al. [8] reported abnormal changes of NAA/Cr (or NAA/Cho) and Cho/Cr. But only the latter calculated absolute concentration and made their results comparable to ours. Phan et al. claimed that the Cr level was not different between groups, thus the change of NAA/Cr (elevated in ACC and reduced in occipital cortex) and Cho/Cr (reduced in ACC and remained unchanged in occipital cortex) can represent the change of NAA and Cho levels. And they speculated that the change of NAA might reflect neuronal reorganization. Given that different VOIs have diverse changes (as demonstrated by Phan et al. [8] ), our arguments about NAA and Cho may derive from the difference of VOI. However the findings of both studies need to be replicated before any convincing conclusion can be make.

All the significant changes revealed in the present study involved the left DLPFC region. To the best of our knowledge, it is the first report of the DLPFC chemistry in patients with SAD. The DLPFC plays an essential role in mood regulation and integration of cognitive functions [41]. The DLPFC has wide reciprocal connections to the limbic structures and mediates the fear extinction by inhibiting the amygdala via ventromedial prefrontal regions [42]. Both morphological and functional changes have been found in DLPFC under various mental disorders [43]–[46]. In addition, the magnitude of increased activation of the left DLPFC is found to be correlated with the emotion regulation [47]. Consistently, we noted a significantly negative linear correlation between Cr concentration of the left DLPFC and the LSAS score. This indicates that Cr level may reflect the symptom severity of SAD. Our findings provide further evidence that deficits in DLPFC function may result in the emotional dysregulation [46].

The major limitation of this study is the limited number of subjects which make us maybe not able to find the difference in other VOIs. Further study with larger sample size may help to confirm our results. In addition, metabolite concentrations in this study is slightly higher than the reported data, possibly due to the contribution from macromolecules at short TE. Studies using techniques like ‘metabolite-nulling’ [48] should be conducted to evaluate their effect. We did not calculate tissue-specific metabolite concentration. However, previous studies have been able to calculate metabolite concentration of ‘pure’ gray or white matter using multi-voxel spectroscopy and tissue segmentation based on high resolution images [49], [50]. Although this method requires longer acquisition time and sacrifices the difference of several voxels, it may provide further insight into the underlying psychopathology of SAD.

In summary, Cr level decreased in the left DLPFC of SAD patients; therefore using absolute metabolite concentration would be a better strategy than using metabolite/Cr peak area ratio for the observation of individual metabolite changes. Decreased Cr level may result from a hypermetabolic state of the left DLPFC and is able to reflect the disease severity.
